# Reply to ‘Increased food supply mitigates ocean acidification effects on calcification but exacerbates effects on growth’

**DOI:** 10.1038/s41598-018-27670-0

**Published:** 2018-06-28

**Authors:** Laura Ramajo, Iris E. Hendriks, Nelson A. Lagos, Dorte Krause-Jensen, Núria Marbà, Mikael K. Sejr, Carlos M. Duarte

**Affiliations:** 1 Department of Sciences, Faculty of Liberal Arts, Universidad Adolfo Ibáñez, Avda. Diagonal Las Torres 2640, Peñalolén, 7941169 Santiago Chile; 2 Centro de Investigación e Innovación para el Cambio Climático (CiiCC), Universidad Santo Tomás, Avda. Ejército 146, 8370003 Santiago, Chile; 3 Center for the Study of Multiple Drivers on Marine Socio-Ecological Systems, Universidad de Concepción, Concepción, Chile; 4 Global Change Department, Instituto Mediterráneo de Estudios Avanzados (IMEDEA, CSIC-UIB), C/Miquel Marqués 21, 07190 Esporles, Islas Baleares Spain; 5 Department of Bioscience, Aarhus University, Vejlsøvej 25, 8600 Silkeborg, Denmark; 6 Arctic Research Centre, Bioscience, Arhus University, C. F. Møllers Allé 8, 8000 Åarhus, Denmark; 7 Red Sea Research Center, King Abdullah University of Science and Technology, Thuwal, 23955-6900 Saudi Arabia

## Abstract

In the Brown *et al*. study ‘Increased food supply mitigates ocean acidification effects on calcification but exacerbates effects on growth’ they show disagreement with the tested hypothesis and data analysis methodology used in our 2016 study. We acknowledge careful criticism and a constructive dialogue are necessary to progress science and address these issues in this reply.

Replying to: Brown *et al*. *Sci. Rep*. **8** (2018); 10.1038/s41598-018-28012-w.

## Introduction

In our 2016 article in *Scientific Reports*^1^, we described the first meta-analysis synthesizing the results of experimental studies that to date had evaluated the role of food availability on susceptibility to ocean acidification of calcifying species. Our results supported the hypothesis that under high food availability, marine calcifiers are capable to mitigate the negative impacts of ocean acidification (OA). The re-examination of our study offered by Brown *et al*.^2^ presents a re-analysis of our results using a different methodological framework to, finally, offer similar results and conclusions as we achieved, with minor deviations despite issuing criticisms to our approach.

The main assertions of Brown *et al*.^2^ are based on:

1. We test a different hypothesis than the one we proposed. We did not. We evaluated how OA affected responses across a range of food availability. Indeed, Brown *et al*.’s^2^ results are consistent with ours, showing that negative effects of OA on calcification were alleviated by high food supply and, also, food supply leads to increased growth under OA scenarios and ambient conditions.
2. Our analysis used inadequate methodology (control treatment selection) by comparing both the high food acidified treatment and the low food acidified treatment to a common reference treatment (high food-non acidified treatment). It is correct that the use of “ambient CO_2_+ high/ambient food supply” as a common control hampers the exact attribution of OA, starvation or their combination as driver for the low calcification response and the low growth response in the low- and intermediate food-supply treatments. In these cases, we can conclude that simultaneous exposure to OA and food deprivation has a large negative impact relative to well-fed, non-acidified controls”. For the “high food-supply treatments” (where the control and treatment can only differ in terms of OA) we can conclude that there is no significant effect of OA on calcification or growth when food is supplied. In our study, we pose the question of how organisms are affected by OA under different food scenarios, and clearly show that potentially negative responses to OA were alleviated when food was supplied as opposed to the situation where the organisms were starved. This conclusion is consistent with that of Brown *et al*., despite the difference in approach to the analysis and the data sets used, indicating that this is indeed a robust finding.
  The decision of what treatments to use as control conditions was based on, with one exception^3^, the fact that studies incorporated in the meta-analysis did not define “control food conditions” properly. Hence, a standardization was necessary to enable comparisons, which was provided by selecting a common condition (with higher food) as control or reference condition.
3. Errors we committed when extracting data from selected studies could have determined our results and conclusions. Although Brown *et al*.^2^ indeed detected some mistakes (but far fewer than they claim), Brown and colleagues^2^ re-analyzed our data, after correcting these minor mistakes, to reach similar results and conclusions as those in our article. Hence, these errors did not affect the conclusions.

Thus, we believe that the re-analysis and methodology used by Brown *et al*.^2^ does not refute our original findings but expands the hypothesis and implications in studies focused on the effects of OA in combination with other stressors, as well as the interactive or additive nature of these effects. Their approach also highlights the complexities involved in estimating univariate measurements (effects sizes) from factorial experimental designs. However, without deepening the potential bias due to the still reduced number of studies^4^ (sample size) and dependence between variables used in any meta-analysis studies, the scope of the Brown *et al*.^2^ approach must be considered with caution in light of their conclusions. In particular, the statement that food would exacerbate the effects of OA on growth responses seems exaggerated. In their discussion Brown *et al*.^2^ neglect that, in most cases, calcification is more negatively affected under OA than growth^5^. Indeed calcification is an energetically-expensive biological process^6^, and maintaining calcification rates in the presence of food limitation imposes physiological trade-offs on other biological features^7^ (e.g., lower growth rates to maintain normal calcification rates and shell functionality).

Finally, we want to highlight that the low sample size in both studies^1,2^ implies that further efforts to experimentally resolve the role of food supply on responses to OA conditions are required to conclusively resolve this important question.
